# Graphene‐Based MicroRNA Transfection Blocks Preosteoclast Fusion to Increase Bone Formation and Vascularization

**DOI:** 10.1002/advs.202102286

**Published:** 2021-08-04

**Authors:** Ce Dou, Ning Ding, Fei Luo, Tianyong Hou, Zhen Cao, Yun Bai, Chuan Liu, Jianzhong Xu, Shiwu Dong

*Adv. Sci*. **2018**, *5*, 1700578

DOI: 10.1002/advs.201700578

In the originally published article, there is an error in Figure 3c, in which the representative bone slice bone resorption images were incorrect. The corrected figure is shown below.

**Figure 3:**
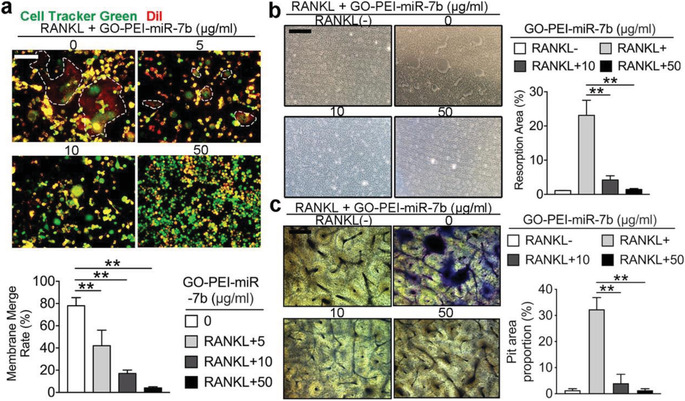
GO‐PEI‐miR‐7b inhibits OC cell–cell fusion and bone resorption. a) Cell–cell fusion assay of BMMs in different groups and quantification of membrane merge rate. b) Pit formation assay images and quantification of resorption area on osteosurface. c) Pit formation assay images and quantification of resorption area on bovine bone slice. Images are representative of *n* = 3 independent experiments. The data in the figures represent the averages ± SD. Significant differences are indicated as ** (*p* < 0.01).

The authors apologize for this error and for any inconvenience caused.

